# Supplementary figures and images for: Identification of Gene Targets for the Sprouting Inhibitor CIPC
Source: Plant Direct. 2025 Apr 9;9(4):e70068. doi: 10.1002/pld3.70068 (PMC11982522; doi:10.1002/pld3.70068)

## Slide 1
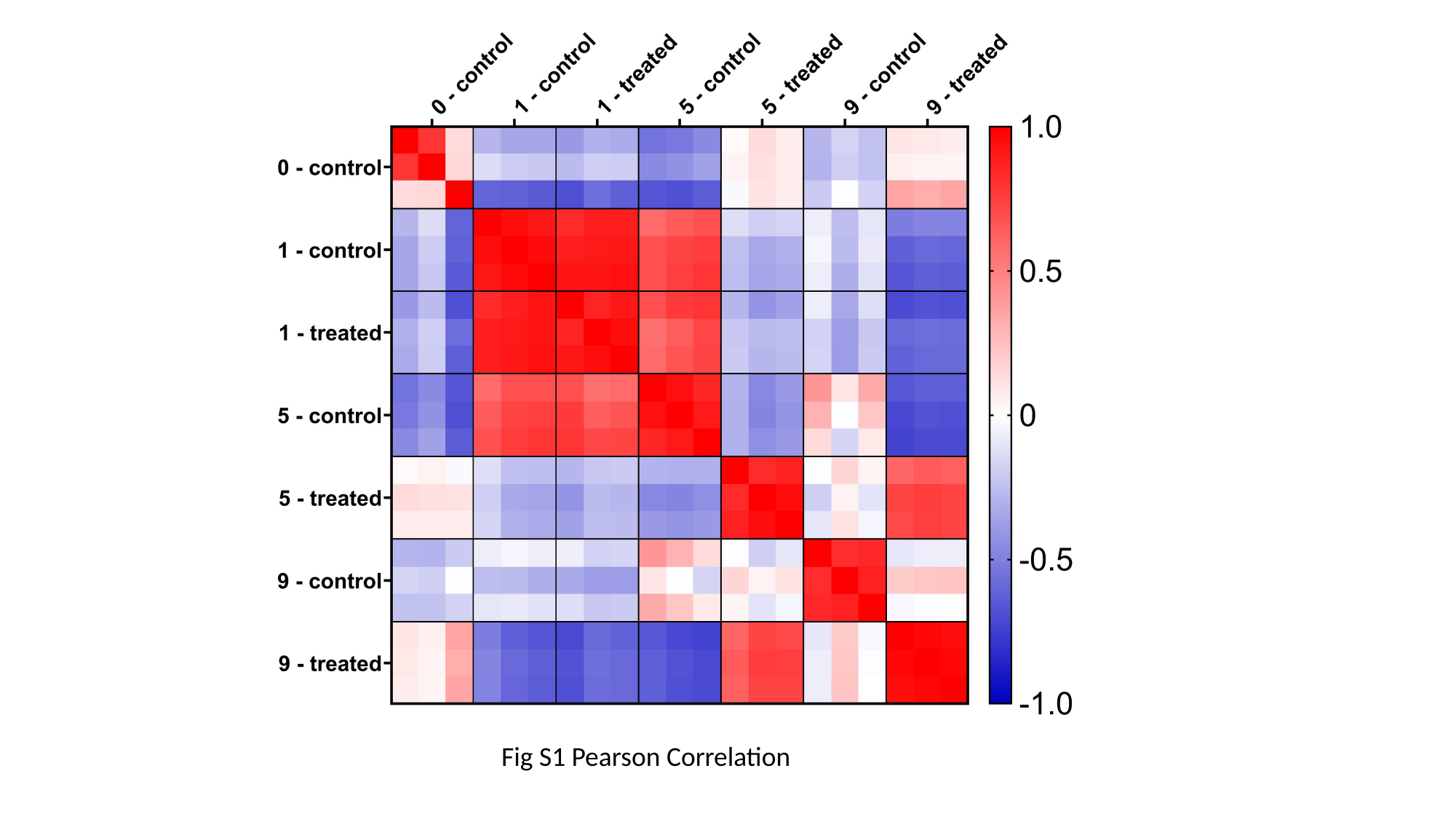

Fig S1 Pearson Correlation

Supplement: Supplementary file 2 — Figure S1 A Pearson's correlation coefficient matrix of RNAseq data shows consistency between most biological replicates. Comparison of all RNAseq samples from CIPC treated and control tubes using a Pearson's correlation coefficient matrix. Red is used to indicate a high level of correlation while blue indicates low correlation between samples. [file PLD3-9-e70068-s001.pptx]
