# Supplementary material for: Identification of Gene Targets for the Sprouting Inhibitor CIPC
Source: Plant Direct. 2025 Apr 9;9(4):e70068. doi: 10.1002/pld3.70068 (PMC11982522; doi:10.1002/pld3.70068)
Supplement: Supplementary file 1 — Data S1 Peer Review [file PLD3-9-e70068-s002.pdf]

Plant Direct
Open Access

Identification of Gene Targets for the Sprouting Inhibitor CIPC

Thomas M Grand, James K Pitman, Alexander L Williams, Lisa M Smith*, Andrew J Fleming*

Decision Letter Round 1:

February 25, 2025

Dr. Lisa M Smith
The University of Sheffield
School of Biosciences
Western Bank
Sheffield, N/A S10 2TN
United Kingdom

RE: Identification of Gene Targets for the Sprouting Inhibitor CIPC

Dear Dr. Smith:

Thank you for submitting to Plant Direct. All required reviews have been returned and we have now finished our evaluation of your manuscript. In light of the reviewers' and editor's comments, further revisions are needed before the paper can be accepted for publication in Plant Direct.

Please view the editors' and reviewers' comments below and use their suggestions as a guide while you work on your revision.

When uploading the revised version of this article, please be sure to include the following:

-A word document that contains your response to the reviewers. You should respond to each reviewer comment and note the changes made to the manuscript. If you do not agree with a reviewer's comment and choose not to make a suggested revision, please explain why. Please try to provide as complete an answer as possible to each reviewer's criticisms.
-A tracked changes document with each change highlighted
- A clean version of the latest version of the manuscript

The reviewers are supportive of the work, and I agree that the manuscript is already acceptable for publication. I am returning for revision to allow the authors to see the comments and perhaps make

minor changes in the discussion.

To upload your revision, please click the link below.
https://plantdirect.msubmit.net/cgi-bin/main.plex?el=A1Lr6mC2A3HIe6I7A9ftdcFGxDOrmbwR1oFwFbpB18gZ

In order to provide as timely a service as possible, we ask that your revision is resubmitted within three months after receipt of this request. If an extension is needed, please send a request, along with a brief explanation, to the editorial office at plantdirect@wiley.com .

Please note that, in addition to publishing reviewer comments, the author's responses to review comments will also be published alongside the final version of the paper. If you would not like the author's responses to be published, please contact the editorial office at plantdirect@wiley.com .

Thank you very much for giving us an opportunity to review your work. I look forward to receiving the next version.

Sincerely,

Gustavo MacIntosh

Editor, Plant Direct

--- Editor comments:
The reviewers are supportive of the work, and I agree that the manuscript is already acceptable for publication. I am returning for revision to allow the authors to see the comments and perhaps make minor changes in the discussion.

--- Reviewer comments:
Reviewer #1:

The manuscript by Grand et al. is an interesting work where the authors have identified the lead gene targeted by the sprouting inhibitor CIPC in potato tuber. It is an important work that has paved the way to develop novel strategies for sprouting inhibition in potato without using pharmacological agents. In this study authors have performed transcriptomics analysis in response to CIPC treatment in time course manner. Furthermore, this has validated some interesting candidate genes involved in cell cycle progression. This study further validates the earlier speculation of CIPC effect on cell cycle during potato

tuber germination. All the experiments are well thought out, carefully designed and beautifully executed. However, I think the manuscripts need further refinement. For example, GO term analysis of RNAseq data clearly shows that the most prominent effect of CIPC treatment is the inhibition of photosynthesis genes. Authors should also clearly explain and further discuss how inhibition of photosynthesis could also affect tuber germination. Another point is that SEM analysis of CIPC-treated sprouts clearly shows a delay in the growth of sprouts without changing much of the developmental trajectory of early meristematic tissue. This delay in germination could also result in reduced cell elongation. I wonder if the author examined the effect of CIPC on cell elongation. For further clarity, I suggest that the author also discusses cell elongation.

Reviewer #2:

This is a well written and accessible paper.
The conclusions are drawn from a combination of physiological observations, transcriptomic analysis and genetic testing. Transcriptomic analysis demonstrates the overall predominance of cell cycle gene regulation in the sprouting response, while the Arabidopsis assay provides evidence of a functional role for this gene. The findings are consistent with previous studies and represent a significant advance in terms of identifying a candidate gene that will allow further understanding of the process of sprouting and sprout repression.

A very minor comment is that as well as being in a different species, the Arabidopsis assay is conducted in a different organ, which I feel that the authors could comment on as it is possible that the developmental niche could affect the role of cell cycle genes. However, the candidate AUG7 is nevertheless strongly identified by this study as have clear importance in the response to CPIC.

Decision Letter Round 2:

March 26, 2025

Dr. Lisa M Smith
The University of Sheffield
School of Biosciences
Western Bank
Sheffield, N/A S10 2TN
United Kingdom

MSID: 2024-01684R1

Plant Direct

Open Access

MS TITLE: Identification of Gene Targets for the Sprouting Inhibitor CIPC

Dear Dr. Lisa Smith:

I am pleased to inform you that your manuscript "Identification of Gene Targets for the Sprouting Inhibitor CIPC" has been accepted for publication in Plant Direct.

Your article will appear online in the next available issue of Plant Direct. To ensure your article gets published as quickly as possible, please pay attention to the steps detailed below. We have found that most of the delays happen at this stage, especially at the payment stage, so please respond as quickly as possible when prompted.

License Agreement: Once your article has been accepted it will move to Production and undergo admin and file checking - you may receive an email with any queries we have at this stage. When all required items are received by the publisher and queries resolved, the corresponding author will receive an email from Wiley's Author Services system which will ask them to log on at https://authorservices.wiley.com/bauthor and will present them with the appropriate license for completion. Your article cannot be published until both the signed license agreement and payment of the article fee have been received.

Payment of the Open Access Article Publication Fee: All articles published in Plant Direct are fully open access: immediately and freely available to read, download and share. Plant Direct charges a publication fee to cover the publication costs. The corresponding author for this manuscript should have already received a quote from the payments team (cs-openaccess@wiley.com) with the estimated article publication fee; please email cs-openaccess@wiley.com if this has not been received. The corresponding author should log on to the Wiley Author Services site, where the publication fee can be paid by credit card or an invoice. Pro Forma can also be requested. Payment of the publication charge must be received before the article will be published online.

Proofs: You will have the opportunity to look over your paper once more when you receive the author proofs for your article. The proofs will be with you in approximately two weeks. Please note that, in addition to publishing reviewer comments, the author's responses to review comments will also be published alongside the final version of the paper. If you would not like the author's responses to be published, please contact the editorial office at plantdirect@wiley.com .

Promotion of your article: You can help your research get the attention it deserves! Check out Wiley's free Promotion Guide for best-practice recommendations for promoting your work at www.wileyauthors.com/eeo/guide. And learn more about Wiley Editing Services which offers professional video, design, and writing services to create shareable video abstracts, infographics, conference posters, lay summaries, and research news stories for your research at www.wileyauthors.com/eeo/promotion.

Thank you again for your contribution to Plant Direct. If you have any questions, feel free to contact the editorial office at plantdirect@wiley.com .

Sincerely,

Gustavo MacIntosh

Editor, Plant Direct

Response to Reviewer:

Response to reviews for 2024-01684:

We thank the reviewers for their considered and positive comments on the manuscript. We believe their comments have helped us to strengthen the discussion and results sections.

Reviewer 1

The manuscript by Grand et al. is an interesting work where the authors have identified the lead gene targeted by the sprouting inhibitor CIPC in potato tuber. It is an important work that has paved the way to develop novel strategies for sprouting inhibition in potato without using pharmacological agents. In this study authors have performed transcriptomics analysis in response to CIPC treatment in time course manner. Furthermore, this has validated some interesting candidate genes involved in cell cycle progression. This study further validates the earlier speculation of CIPC effect on cell cycle during potato tuber germination. All the experiments are well thought out, carefully designed and beautifully executed. However, I think the manuscripts need further refinement.

1. For example, GO term analysis of RNAseq data clearly shows that the most prominent effect of CIPC treatment is the inhibition of photosynthesis genes. Authors should also clearly explain and further discuss how inhibition of photosynthesis could also affect tuber germination.

We thank the reviewer for this suggestion and have expanded our discussion to read:
'There was also a difference in the expression of genes related to photosynthesis, which we interpret as being a consequence of CIPC-related growth retardation leading to disruption of photosynthetic

differentiation. Inhibition of photosynthetic competency may further contribute to suppression of sprouting through limiting photosynthates available for further growth of the bud. During normal sprouting visible greening of the sprout occurs as it extends towards the light. This did not occur in the CIPC-treated buds.'

2. Another point is that SEM analysis of CIPC-treated sprouts clearly shows a delay in the growth of sprouts without changing much of the developmental trajectory of early meristematic tissue. This delay in germination could also result in reduced cell elongation. I wonder if the author examined the effect of CIPC on cell elongation. For further clarity, I suggest that the author also discusses cell elongation.

Interestingly, after noticing that some CIPC-treated buds appeared to have increased cell surface areas, we indeed examined cell size based on area of epidermal cell. However, the increase in cell size in CIPC-treated buds seemed to be stochastic, with some buds having cell sizes equivalent to control untreated buds, and some buds having cell areas at least twice as large. Since there was such variability between buds even within an experiment, and as we were unable to pinpoint the underlying cause of this variability, we elected to exclude this result from the paper. However, the data suggest that cell elongation/expansion is probably not the direct target of the observed phenotype.

Since all prior studies of CIPC function have indicated that growth is limited through changes in mitosis, as discussed in the introduction of the manuscript, and cell elongation was not picked up in our GO term analysis, we think it is unlikely that CIPC affects cell elongation as a primary mechanism.

3. Figure 1 is not labeled correctly. Please correct the labeling. Figure legend mentions alphabets, but it is missing in figure panel.

We have made the letters on each panel bold to ensure they are more visible.

4. Please provide statistics for Fig. 1. How many tubers were analyzed and how many show phenotype.

Four replicates were used for each condition in this experiment, with all buds at the concentration of 100 uM CIPC showing strong growth repression. This experiment was one of multiple experiments performed by two independent PhD students which showed consistent results. As this is presented as qualitative data, statistics are not appropriate.

5. In fig 5, graphs are difficult to read. Please provide better graphs with readable X and Y axis.

We have increased the font size for the axes labels to improve readability.

Reviewer 2

This is a well written and accessible paper.
The conclusions are drawn from a combination of physiological observations, transcriptomic analysis and genetic testing. Transcriptomic analysis demonstrates the overall predominance of cell cycle gene regulation in the sprouting response, while the Arabidopsis assay provides evidence of a functional role for this gene. The findings are consistent with previous studies and represent a significant advance in terms of identifying a candidate gene that will allow further understanding of the process of sprouting and sprout repression.

1. A very minor comment is that as well as being in a different species, the Arabidopsis assay is conducted in a different organ, which I feel that the authors could comment on as it is possible that the developmental niche could affect the role of cell cycle genes. However, the candidate AUG7 is nevertheless strongly identified by this study as have clear importance in the response to CPIC.

We thank the author for this suggestion and have added the following into the manuscript:

'We have interpreted the reduction in root growth in Arabidopsis as CIPC inhibiting cell division. Previous studies on wheat and onion root tips have indicated that CIPC acts via mitosis regardless of organ or species (Eleftheriou and Bekiari 2000; Vaughn and Lehnen 1991), although it is conceivable that CIPC may target different but related cell cycle genes depending on species and developmental niche.'
